# Plantar Pressure Distribution in Charcot–Marie–Tooth Disease: A Systematic Review

**DOI:** 10.3390/s25144312

**Published:** 2025-07-10

**Authors:** Alberto Arceri, Antonio Mazzotti, Federico Sgubbi, Simone Ottavio Zielli, Laura Langone, GianMarco Di Paola, Lorenzo Brognara, Cesare Faldini

**Affiliations:** 1 Department of Biomedical and Neuromotor Sciences (DIBINEM), University of Bologna, 40127 Bologna, Italy; alberto.arceri@ior.it (A.A.); lorenzo.brognara2@unibo.it (L.B.); cesare.faldini@ior.it (C.F.); 2 1st Orthopaedics and Traumatologic Clinic, IRCCS Istituto Ortopedico Rizzoli, 40136 Bologna, Italy; federico.sgubbi@ior.it (F.S.); simoneottavio.zielli@ior.it (S.O.Z.); laura.langone@ior.it (L.L.); gianmarco.dipaola@ior.it (G.D.P.)

**Keywords:** Charcot–Marie–Tooth, plantar pressure, baropodometry, in-shoe sensors

## Abstract

*Background*: Charcot-Marie-Tooth (CMT) disease is a hereditary motor and sensory neuropathy that affects foot morphology and gait patterns, potentially leading to abnormal plantar pressure distribution. This systematic review synthesizes the existing literature examining plantar pressure characteristics in CMT patients. *Methods*: A comprehensive search was conducted across PubMed, Scopus, and Web of Science databases. Risk of bias was assessed using the Newcastle–Ottawa Scale. *Results*: Six studies comprising 146 patients were included. Four studies employed dynamic baropodometry, and two used in-shoe pressure sensors to evaluate the main plantar pressure parameters. The findings were consistent across different populations and devices, with a characteristic plantar-pressure profile of marked midfoot off-loading with peripheral overload at the forefoot and rearfoot, often accompanied by a lateralized center-of-pressure path and a prolonged pressure–time exposure. These alterations reflect both structural deformities and impaired neuromuscular control. Interventional studies demonstrated a load redistribution of pressure after corrective surgery, though residual lateral overload often persists. *Conclusions*: Plantar pressure mapping seems to be a valuable tool to identify high-pressure zones of the foot in order to personalize orthotic treatment planning, to objectively monitor disease progression, and to evaluate therapeutic efficacy. Further longitudinal studies with standardized protocols are needed to confirm these results.

## 1. Introduction

Charcot–Marie–Tooth (CMT) disease is among the most common inherited neurological disorders with an estimated prevalence of 17–40 cases per 100,000 individuals [1,2]. It is an inherited sensory–motor neuropathy of the peripheral nervous system. Patients typically present with asymmetrical, slowly progressive distal motor neuropathy, which most often manifests between the first and third decades of life and leads to muscle weakness and atrophy [3]. Clinical and neurological examinations, aimed at identifying trophic alterations and muscle imbalance, are essential for the recognition of CMT. Electromyography provides supportive evidence for the diagnosis, which can be definitively confirmed through molecular genetic analysis [4,5,6] and subsequently classified according to one of the known genotype-specific subtypes [1,6]. CMT often leads to foot deformities such as cavo-varus, secondary to peripheral muscle imbalance, that increases in severity and rigidity over time. The foot deformity is often associated with pressure lesions, metatarsalgia, and difficulty with shoe fitting [3,7]. The characteristic gait in CMT is marked by an exaggerated high-stepping pattern, terminating in either a toe-to-heel or flat-foot loading response rather than the normal heel-to-toe sequence. These aberrant gait biomechanics primarily reflect inadequate dorsiflexion during the swing phase due to tibialis anterior weakness and altered proprioception [8,9]. These alterations in gait mechanics can significantly compromise mobility, increase fatigue, and reduce quality of life, leading to functional limitations and social participation restrictions [9].

Plantar pressure analysis provides a non-invasive method to quantify foot–ground interaction during static stance or dynamic walking [10,11,12]. In orthopedic practice, pressure mapping informs the management of diverse conditions, including hallux valgus [13,14,15], pes planus [16,17], diabetic foot [18,19,20], and osteoarthritis [21,22,23]. By identifying high-pressure zones and temporal asymmetries, clinicians could tailor orthotic devices, select surgical procedures, and monitor rehabilitation efficacy. In CMT, such analyses may elucidate specific compensatory mechanisms and biomechanical impairments [24]. Despite its clinical relevance, literature on plantar pressure distribution in CMT remains poorly studied. This systematic review aims to summarize the available evidence from studies on plantar pressure profiles in CMT patients and evaluate the characteristics of pressure mapping.

## 2. Materials and Methods

### 2.1. Search Strategy

A comprehensive search of the existing literature pertaining to plantar pressure distribution in CMT patients was performed across three prominent databases: PubMed, Scopus, and Web of Science. The search strategy combined the following keywords: “Charcot-Marie-Tooth” AND (plantar pressure distribution OR baropodometry OR in-shoe sensors OR gait analysis). All keywords were meticulously examined both individually and combined with their Medical Subject Heading (MeSH) terms. The search included all studies published until 1 May 2025. Reference lists of relevant articles were also screened manually. Two authors (G.D.P. and F.S.) independently conducted the literature search. The protocol was registered in the PROSPERO database (Registration ID: CRD420251053747). This systematic review was conducted according to the PRISMA 2020 guidelines [25].

### 2.2. Inclusion and Exclusion Criteria

Original observational studies involving patients with genetically or clinically confirmed CMT reporting the assessment of plantar pressure using validated tools (e.g., baropodometry, in-shoe systems) were included. Exclusion criteria included review articles, conference abstracts, case reports, editorials, studies not measuring plantar pressure directly, and non-English articles.

### 2.3. Study Selection and Data Extraction

After duplicates removal, two authors (A.A. and F.S.) independently conducted an assessment of the eligibility of papers by scrutinizing their titles and abstracts, in accordance with predefined inclusion and exclusion criteria. Subsequently, relevant full-text articles were retrieved for eligibility. Disputes regarding inclusion of an article were resolved from the senior author (C.F.).

Data extracted included: authorship, publication year, study design, sample size characteristics, pressure measurement technique and device used, measured parameters, and key findings. Data extracted from these studies were organized based on the participants, intervention, comparisons, and outcomes (PICO) question and includes:

- Participants: number of patients and demographic characteristics (age and CMT subtype);
- Intervention: measurement method (baropodometry, in-shoe sensors) and devices used;
- Comparisons: comparison of the plantar pressure main findings between healthy controls and unaffected feet, idiopathic cavus vs. neurogenic foot, pre- and post-operative period;
- Outcomes: plantar pressure parameters.

Data collection was performed using Microsoft Excel (Microsoft Corporation, Redmond, WA, USA) for Windows 11.

### 2.4. Risk of Bias Assessment

Quality assessment for the selected studies was evaluated by two independent reviewers (G.D.P. and F.S.) using the Newcastle–Ottawa Scale (NOS) [26]. This scale assesses eight domains. A total score out of 9 was assigned to each study, with ≥5 stars indicating low risk and ≤4 high risk.

### 2.5. Data Analysis

A descriptive analysis was performed to report major findings, and no meta-analysis was planned as intervention diversity, comparators, and outcomes were expected. Data from the included studies were reported using textual descriptions of outcome measures, based on the original authors’ presentation of statistically significant findings.

## 3. Results

**Figure 1:**
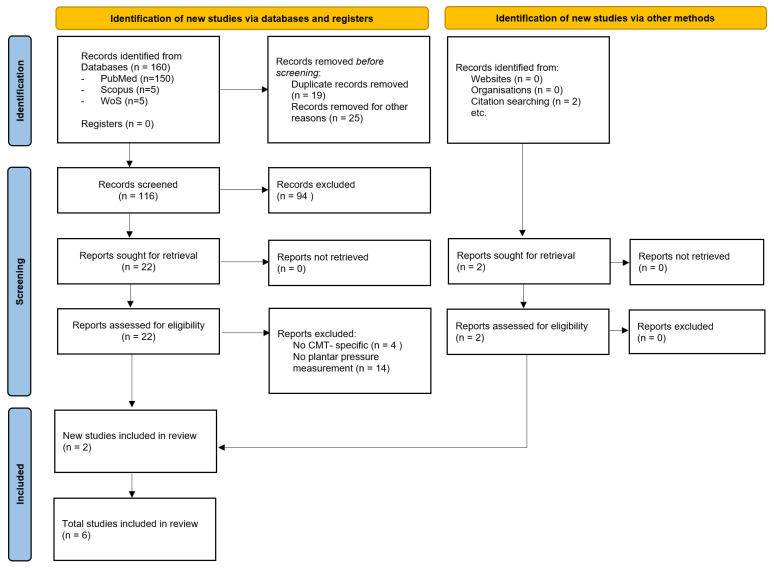
PRISMA flowchart for selection of the studies [27].

From an initial yield of 160 records, 19 duplicates were removed. Title and abstract screening excluded 119 articles, leaving 22 for full-text review. Additionally, 2 relevant studies were identified through manual screening of the reference lists of the included articles. Thus, 6 articles were found to meet the selection criteria and were included in the qualitative synthesis. The process of selection was reported in Figure 1. Detailed data regarding the included studies is provided in Table 1.

### 3.1. Risk of Bias

The quality assessment of the included studies is reported in Table 2. All studies achieved NOS scores of 7 considered low risk. The included seven studies spanned 2005–2023, including cross-sectional and interventional designs (Table 1).

### 3.2. Population

The included seven studies comprised 146 CMT patients, with a mean age of 26.3 ± 17.3 years, ranging from 10 to 50 years (Table 1).

CMT subtypes were variably reported (Table 1), specifically CMT1A predominated in pediatric cohorts [28,30,32,32,33] accounting for 62.3% of cases of selected studies, while adult case series included mixed hereditary motor–sensory neuropathy (HMSN) presentations [28,29,31,33].

### 3.3. Plantar Pressure Systems and Outcomes

Most studies (n = 4) employed dynamic baropodometry, whereas in-shoe systems were utilized in two studies. Specific devices are reported in Table 1.

The main plantar pressure parameters that were reported in the studies included in this review are listed below:

- Peak pressure (PP) is the maximum pressure recorded under a defined plantar region during the stance phase, typically expressed in kilopascals (kPa). PP reflects the highest localized load experienced by the foot [34].
- Pressure–time integral (PTI) is the area under the pressure–time curve for a given region, combining the magnitude and duration of loading (units kPa·s). PTI provides a cumulative measure of tissue stress throughout stance and may be more sensitive to overall load exposure than PP alone [35].
- Center-of-pressure (CoP) trajectory is the path traced by the centroid of the resultant ground reaction force vector across the plantar surface during stance. CoP trajectory characterizes dynamic balance control and gait progression, with deviations indicating altered foot function or stability [36].
- Contact time (CT) is the total duration for which the foot remains in contact with the support surface during a single gait cycle stance phase, usually measured in seconds. CT reflects temporal aspects of gait and is prolonged in conditions with muscle weakness or neuromuscular impairment [37].
- Contact area (CA) is the surface area of the plantar foot in contact with the sensor or platform at any instant, reported in square centimeters (cm^2^). CA indicates the extent of load distribution under the foot and is altered in deformities that restrict or expand regional contact [38].
- Peak force is the highest resultant force applied to a plantar region during stance, often normalized to body weight (N·%BW). Peak force quantifies the maximum load transmitted through the foot and, together with CA, underpins the calculation of PP [33].
- Root mean square deviation (RMSD) is a statistical measure of the average deviation of an individual’s plantar pressure map from a normative reference pattern. RMSD captures global abnormalities in pressure distribution and is elevated in neuropathic or structural foot disorders [33].

**Figure 2:**
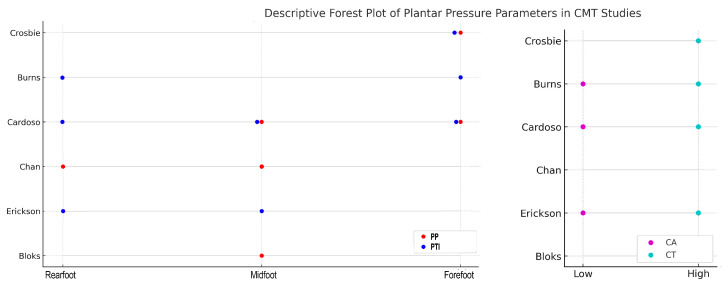
Descriptive forest plot summarizing plantar pressure parameters reported across six studies on Charcot–Marie–Tooth disease.

Despite heterogeneity in instrumentation and gait conditions, across all studies, CMT-related cavovarus feet displayed a hallmark pressure pattern; there was a pronounced off-loading of the midfoot throughout stance while the rearfoot and forefoot underwent significant pressure [28,29,30,31]. This translates into elevated PP, PTI, and CT exposure at these peripheral sites, especially under the forefoot [28,29,30]. Several studies have also documented significant elevated pressures at the lateral midfoot and rearfoot [30,31,32], which are corroborated by a corresponding lateral deviation of the CoP trajectory [33]. Most studies reported a reduction in CA, thus confirming the presence of the hallmark of the cavus arch [29,30,31,32]. Muscle weakness and foot-drop slow gait progression, leading to a lengthening of the stance phase and uniformly sustained pressure increase across the plantar surface rather than isolated peaks [28,29,30,32,33]. For instance, adolescents show near-normal PP compared to children but typically exhibit longer stance durations relative to controls, highlighting the lingering influence of neuromuscular factors [30] (Figure 2).

Surgical interventions partially restore more normal load distribution, with some studies reporting improved midfoot engagement and reduced forefoot spikes, though residual lateral overload often persists [31,32].

## 4. Discussion

This systematic review consolidates current knowledge on plantar pressure abnormalities in CMT patients. The findings were consistent across different populations and devices, reporting bi-point overloading at the forefoot and rearfoot level and reduced medial foot loading. This was occasionally accompanied by lateralized pressure patterns. These alterations reflect both structural deformities and impaired neuromuscular control.

The CMT population considered in this review was predominantly young and also comprised different subtypes. CMT1A is a predominantly demyelinating neuropathy accounting for the majority of CMT cases and involves mainly pediatric patients [39]. This condition, caused by PMP22 gene duplication, typically presents with mild to moderate distal muscle weakness, areflexia, sensory loss, and cavus foot [2,40,41,42]. The prevalence of other CMT subtypes was reported with a high degree of variability [43]. CMT2 involves axonal degeneration with later onset (adolescence to early adulthood) and variable severity, frequently manifesting as a milder form compared to CMT1 [44]. Unclassified HMSN groups often reported heterogeneous sensory–motor deficits. Unspecified HMSN denotes those pedigrees exhibiting either demyelinating or axonal electrophysiological profiles without identifiable mutations in common CMT genes; such cases present with the same length-dependent motor and sensory deficits, foot deformities, and hypo- or areflexia seen in CMT1/2 [45,46].

Two main measurement systems are currently employed for plantar pressure assessment: pedobarographic platforms and in-shoe sensor systems [14,47,48,49,50]. Both utilize electronic pressure transducers to record plantar pressure distribution under static and dynamic conditions [11,38]. Pedobarographic platforms are rigid, floor-embedded platforms consisting of an array of pressure sensors aligned parallel to the walking surface. Spatial resolution depends on sensor density, as specified by each manufacturer. Platforms are well-suited for laboratory-based analyses of standing posture and simple gait tasks, offering precise mapping of plantar pressures. However, they require sufficient floor space and subject gait consistency to ensure full contact with the sensing area [51]. In-shoe sensor systems consist of thin, flexible sensor sheets placed between the foot and the shoe interior. Constructed from multi-layer polyester substrates with printed electronic circuits, in-shoe systems vary in sensor thickness and density. The in-shoe system is characterized by its portability, which facilitates ambulatory monitoring of pressure distribution across a range of gait activities, footwear configurations, and terrains. In contrast, the platform necessitates a more expansive spatial requirement for measurement and patient’s ability to contact the platform [51]. The key limitation of in-shoe sensors is lower spatial resolution compared to platform devices, owing to a reduced sensor count per unit area [51]. Both approaches support a variety of pressure transducer technologies, including capacitive, resistive, piezoelectric, and piezo-resistive sensors, permitting choice based on accuracy, flexibility, and application requirements [51]. The variability in plantar pressure measurement approaches across studies likely influenced the comparability of the reported outcomes. Baropodometric platforms, while offering higher spatial resolution and stability under controlled laboratory conditions, may fail to capture dynamic in-shoe forces during real-life ambulation. In-shoe sensor systems often lack sensitivity in detecting medio-lateral force components and may be affected by shoe type and insole fit. These methodological differences may partly account for the heterogeneity observed in plantar pressure parameters across studies. Therefore, standardization of measurement protocols and reporting formats is essential for future research.

The plantar pressure pattern is due not only to the morphology of the foot but also to dynamic muscle control [7,28]. A normal foot distributes load smoothly along the arch, while a cavus foot concentrates pressure beneath the rearfoot and forefoot [29]. In neurogenic cavus, such as that seen in CMT disease, peripheral neuropathy induces two additional characteristic alterations. First, selective weakness of dorsiflexors (tibialis anterior) and evertors (peroneus brevis), coupled with relatively preserved invertors (tibialis posterior and peroneus longus), produces progressive hindfoot inversion and accentuated lateral loading along the midfoot [52]. Second, gait mechanics are fundamentally altered [53]; instead of a heel strike–foot flat–heel off pattern during walking, CMT patients often initiate ground contact with the forefoot, either simultaneously with or prior to the heel, thereby prolonging the stance phase, raising pressure and contact time across all foot regions, and decreasing pressure under the rearfoot compared to idiopathic cavus [29,53]. This presentation could be explicable in terms of the neuropathic changes and their effects. If the heel strike is absent or minimal, rearfoot pressure may be expected to decrease [28].

Dynamic muscle control is crucial also when considering surgical corrections. In some interventional studies altered preoperative plantar pressure pattern was partially improved postoperatively [31,32]. In one study, the lateral midfoot pressures normalized toward control values and medial midfoot pressures decreased further, but rearfoot pressures increased while forefoot pressures remained lower than normal data [31]. Similarly, postoperative measurements in another study showed a generalized increase in contact area and decrease in peak force, reflecting a partial redistribution of load toward a more physiological pattern, though some residual lateral overload persisted [32]. Although radiographic parameters corrected to near-normal, yet pedobarographic redistribution was incomplete. The lack of heel-strike normalization—evidenced by persistently elevated heel pressures inversely correlated with ankle power generation—underscores the influence of gastrocnemius–soleus weakness on postoperative gait dynamics. This analysis demonstrates that, although surgical correction realigns osseous deformities, persistent neuromuscular deficits may limit full restoration of physiological plantar pressure patterns in CMT-related cavovarus feet [31]. Interventional studies [31,32] reported combinations of soft-tissue and bony procedures, including plantar fascia release to alleviate medial arch tension, Steindler stripping to rebalance the intrinsic foot muscles, and tendon transfers, such as posterior tibialis transfers to the dorsum and Jones transfers from peroneus longus to peroneus brevis, to correct muscular imbalance. In addition, gastrocnemius recessions were performed, as needed, to address equinus contracture. Metatarsal osteotomies (often of the first ray) were performed to realign the forefoot. Select cases also received midfoot osteotomies for rigid deformities. Nevertheless, the extent to which individual surgical procedures influence postoperative plantar pressure redistribution remains unclear. To date, no study has conducted a formal correlation analysis between specific operative techniques and quantitative changes in plantar pressure parameters. As a result, the relative contribution of each procedure to midfoot engagement, PP reduction, or PTI normalization has yet to be systematically determined. Similar findings have been reported in the context of instrumented gait analysis in children with cerebral palsy, where objective pressure and kinematic data have proven valuable in guiding treatment planning and evaluating surgical outcomes [54]. This parallel highlights the broader applicability and clinical utility of plantar pressure mapping across neuromuscular disorders.

Plantar pressure assessment could have significant clinical value in the management of Charcot–Marie–Tooth disease [55,56]. By delineating regions of excessive loading and off-loading, these measurements can directly inform the customization of orthotic insoles and ankle–foot orthoses to redistribute forces and protect vulnerable tissues [57,58,59,60,61]. Longitudinal pressure mapping enables objective monitoring of disease progression and deformity evolution, facilitating timely intervention [62,63,64]. Furthermore, preoperative pressure profiles can potentially guide the selection and sequencing of surgical techniques, such as tendon transfers or osteotomies, by identifying the deformity’s functional drivers [65]. Finally, standardized plantar pressure parameters may serve as robust, quantifiable endpoints in rehabilitation and orthotic efficacy trials, allowing clinicians to track gait improvements and optimize therapy protocols [49].

The included studies were rated as high quality based on the NOS. However, it is important to note that this does not necessarily reflect overall methodological comprehensiveness, particularly regarding standardization of protocols or outcome interpretation. Notably, none of the included studies reported inter-rater or intra-rater reliability for plantar pressure data, which should be addressed in future work to strengthen reproducibility. Furthermore, a major limitation of the current body of literature is the scarcity of interventional studies specifically designed to alter plantar pressure patterns in individuals with CMT and evaluate their clinical impact. In addition, the overall number of studies available on this topic remains limited by small sample sizes, lack of longitudinal follow-up in most studies, and heterogeneity across age groups, CMT subtypes, and sensor systems and protocols, which may have introduced variability and limited the generalizability of results. These aspects should be addressed in future studies to improve reproducibility and generalizability.

Normative values used for comparison in the included studies were generally derived from age-matched healthy control groups or from validated baropodometric reference databases [66]. Future comparisons should utilize pediatric- or adult-specific reference data accordingly. While these sources provide a useful benchmark for identifying deviations in plantar pressure patterns, it is important to note that variations in equipment calibration, walking protocols, and population characteristics may affect the consistency of such reference data across studies. Nonetheless, the uniformity in observed pressure patterns reinforces the clinical relevance of pressure mapping in CMT management. Despite these limitations, to our knowledge, this is the first systematic review on the topic. This study aimed to increase interest in this less-studied field and provide new insights into the topic in order to help future research to adopt standardized protocols, larger multicenter cohorts, and formal analyses linking specific surgical techniques or orthotic designs to quantitative improvements in plantar pressure metrics.

Future studies should aim at standardizing data acquisition protocols, including minimum gait cycles, sensor density, walking speed, step length normalization, and consistent segmentation of foot regions. Wearable plantar pressure systems represent a promising tool to capture gait patterns during daily activities, potentially overcoming the limitations of lab-based assessments [11]. Advanced pressure mapping tools capable of detecting shear forces and axial deviations (e.g., 3D multi-axis systems) could provide more comprehensive biomechanical profiling. Longitudinal monitoring, ideally on an annual basis using the same calibrated device, would allow for early detection of deformity progression or functional decline, as previously demonstrated in diabetic foot populations [35]. Furthermore, future research should incorporate validated clinical outcome measures (e.g., pain, mobility, function) in parallel with plantar pressure assessment to clarify the clinical impact of observed biomechanical changes. Machine learning and pressure-based predictive analytics may further aid to classify gait alterations and detect early signs of deformity progression in CMT, stratifying surgical or orthotic indications in CMT patients.

## 5. Conclusions

This systematic review confirms that CMT-associated cavovarus deformity produces a characteristic plantar pressure profile of marked midfoot off-loading with peripheral overload at the forefoot and rearfoot, often accompanied by a lateralized center-of-pressure path. These patterns arise from both the rigid, high-arched morphology and the neuromuscular deficits intrinsic to CMT, which alter normal gait mechanics and prolong stance duration. While surgical interventions can partially normalize load distribution, persistent muscle weakness frequently limits full restoration of physiological pressure patterns. Plantar pressure mapping appears to be a promising and valuable tool for personalized orthotic treatment planning, objective monitoring of disease progression, and evaluation of therapeutic efficacy.

## Figures and Tables

**Table 1:** Summary of studies evaluating plantar pressure distribution in Charcot–Marie–Tooth (CMT) patients.

Study (Year)	Study Design	Sample Size (Mean Age)	CMT Subtype	Method (Device)	Measured Parameters	Main Findings
Crosbie et al. (2008) [28]	Cross-sectional	16 (51.7 ± 16.2 y)	CMT1 (9), unknown (4), CMT2 (2), CMT X (1)	In-shoe sensors (Novel Pedar)	PP, PTI, CT	↑ PP and PTI forefoot, prolonged CT
Burns et al. (2005) [29]	Cross-sectional (idiopathic cavus vs. neurogenic foot vs. controls)	10 CMT	Mixed	Dynamic baropodometry (EMED platform)	PP, PTI, CA, CT	↑ PTI rearfoot and forefoot; ↓ CA; prolonged CT
Cardoso et al. (2021) [30]	Cross-sectional (children vs. adolescent vs. controls)	40 (12.7 ± 0.6 y)	CMT1A (22), CMT2A (6), CMTX (1), unknown (11)	In-shoe sensors (Pedar-X)	PP, PTI, CA, CT	Children: ↓ CA, ↑ CT, ↑ PP medial forefoot and midfoot, ↑ PTI rearfoot, lateral midfoot and medial forefoot; Adolescents: ↓ CA, ↑ CT, no difference PP and PTI
Chan et al. (2007) [31]	Prospective interventional	9 (13.3 ± 2.5)	Unspecified CMT	Dynamic pedobarograph (Tekscan High-Resolution Pressure Assessment System)	Segmental pressure–time profiles pre/post-surgery	Pre-op: ↑ lateral midfoot and rearfoot; ↓ medial midfoot and forefoot Post-op: ↓ lateral midfoot and medial midfoot, ↑ rearfoot
Erickson et al. (2015) [32]	Prospective interventional	19 (11 ± 2 y)	CMT1A	Dynamic pedobarograph (EMED platform)	PP, PTI, peak force, CA, CT	Pre-op: ↑ PTI and CT lateral midfoot and rearfoot; ↓ CA and PP medial midfoot Post-op: ↓ peak force, ↑ CA
Bloks et al. (2023) [33]	Case–control	52 (42.7 ± 17.1 y)	CMT1 (41), CMT2 (9), unknown (2)	Dynamic baropodometry (Footscan pressure plate)	RMSD, PP ratios, CoP trajectories	↑ RMSD, ↑ CoP lateral deviation; ↑ PP ratios lateral midfoot

Note: ↓/↑ = decrease/increase compared to controls; CoP = center of pressure; PTI = pressure–time integral; PP = peak pressure; CT = contact time; CA = contact area; RMSD = root mean square deviations; HMSN = hereditary motor sensory neuropathies. Only statistically significant results, as reported by the original studies, are included.

**Table 2:** Risk of bias assessment using the Newcastle–Ottawa Scale.

Study (Year)	Representativeness	Control Selection	Case Definition	No Outcome at Start	Comparability	Assessment Outcome	Follow-Up Long Enough	Outcome Adequacy	Total
Crosbie et al. (2008) [28]	★	★	★	★	★	★	—	★	**7** ★
Burns et al. (2005) [29]	★	★	★	★	★	★	—	★	**7** ★
Cardoso et al. (2021) [30]	★	★	★	★	★	★	—	★	**7** ★
Chan et al. (2007) [31]	★	—	★	★	★★	★	—	★	**7** ★
Erickson et al. (2015) [32]	★	—	★	★	★★	★	—	★	7 ★
Bloks et al. (2023) [33]	★	★	★	★	★	★	—	★	**7** ★

*Note*: The total quality score ranged from 0 to 9, with studies scoring ≥ 5 points being considered at low risk of bias, while those scoring < 5 points were classified as high risk of bias. A study can be given a maximum of one star for each numbered item within the selection and outcome categories. A maximum of two stars can be given for comparability.

## Data Availability

The data presented in this study are available on request from the corresponding author; however, restrictions apply to the availability of these data, which were used under license for this study, and they are thus not publicly available.

## Abbreviations

The following abbreviations are used in this manuscript:

CMT	Charcot–Marie–Tooth
NOS	Newcastle–Ottawa Scale
HMSN	Hereditary Motor Sensory Neuropathies
PP	Peak Pressure
PTI	Pressure–time integral
CoP	Center-of-pressure
CA	Contact area
CT	Contact time
RMSD	Root mean square deviation
